# Metabolic syndrome and cognitive deficits in the Greek cohort of Epirus Health Study

**DOI:** 10.1007/s10072-023-06835-4

**Published:** 2023-05-10

**Authors:** Myrto Koutsonida, Fotios Koskeridis, Georgios Markozannes, Afroditi Kanellopoulou, Abdou Mousas, Evangelos Ntotsikas, Panagiotis Ioannidis, Eleni Aretouli, Konstantinos K Tsilidis

**Affiliations:** 1 Department of Hygiene and Epidemiology, School of Medicine, University of Ioannina, Ioannina, Greece; 2 B’ Department of Neurology, AHEPA University Hospital of Thessaloniki, Aristotle University of Thessaloniki, Thessaloniki, Greece; 3 Department of Psychology, School of Social Sciences, University of Ioannina, Ioannina, Greece; 4 Department of Epidemiology and Biostatistics, School of Public Health, Imperial College London, London, United Kingdom

**Keywords:** metabolic syndrome, components, cognition, attention, memory

## Abstract

**Background:**

Metabolic syndrome is considered an important risk factor for cognitive decline and dementia. However, the evidence in middle-aged individuals is still conflicting. The aim of the study was to explore the association between metabolic syndrome and its individual components with cognitive function and to investigate possible interaction between sex, age and genetic predisposition for metabolic syndrome and Alzheimer’s disease in a middle-aged Greek cohort.

**Methods:**

A total of 2,077 healthy adults (mean age: 46.7 years) were included in the primary cross-sectional analysis and 305 of them in secondary prospective analyses. Metabolic syndrome was defined by the revised National Cholesterol Education-Adult Treatment Panel III and the International Diabetes Federation criteria. Cognitive function was measured primarily with the Trail Making, Verbal fluency and Logical Memory test, and in secondary prospective analyses with online versions of Posner cueing task, an emotional recognition task, Corsi block-tapping task and Stroop task.

**Results:**

Multivariable linear regressions showed an association of metabolic syndrome with lower performance in attention (β=1.62 seconds, 95% CI=0.20, 3.04) and memory (β=-0.62 words, 95% CI=-1.19, -0.05) that could be driven by associations with elevated fasting glucose and abdominal obesity. Similar associations were observed in the secondary prospective analyses.

**Conclusion:**

In summary, metabolic syndrome was associated with cognitive deficits in domains related with the cognitive profile of vascular cognitive impairment.

**Supplementary Information:**

The online version contains supplementary material available at 10.1007/s10072-023-06835-4.

## Introduction

Metabolic syndrome (MetS) is a constellation of four cardiometabolic disorders - elevated blood pressure, glucose intolerance, dyslipidemia and abdominal obesity- that is associated with increased risk of cardiovascular diseases. Although there are conflicts among clinicians regarding the clinical utility of the term “metabolic syndrome” [1–3], the interest in public health research is growing since metabolic syndrome as a clinical entity highlights the multifactorial aspect of the disease state.

Different criteria for the definition of MetS have been proposed by several health organizations including World Health Organization (WHO) [4], European Group for the study of Insulin Resistance (EGIR) [5], National Cholesterol Education-Adult Treatment Panel III (NCEP-ATP III) [6], American Heart Association (AHA) [7] and International Diabetes Federation (IDF) [8]. As a result, the global prevalence of MetS varies from 12.5% using the NCEP-ATP III criteria to 29.1% using the AHA criteria [9]. These percentages indicate that over a billion of people worldwide suffer by MetS, supporting that MetS is a major public health issue.

Interestingly, MetS has been previously reported by several studies to present a protective role on cognition in individuals older than 75 years [10–12]. However, the association of MetS on cognition is not yet well-established in middle-aged or people younger than 75 years. There are studies that reported an aggravating impact of MetS on cognitive domains [13–16], whereas other studies reported no associations [17–19].

Some recent reviews have attempted to summarize the results from studies that examined the association of MetS with cognition [20–25], but most of them remained inconclusive and stated the need for more research with standardized neuropsychological tests. The aim of the present study was to investigate the cross-sectional association of MetS and its individual components with cognitive abilities in a mostly middle-aged Greek cohort using widely administered neuropsychological tests with available normative data for the Greek population. Moreover, we sought to replicate these possible associations in a smaller group of participants that completed an online neuropsychological examination relatively close after recruitment. Finally, we investigated possible pairwise interactions by sex and age as indicated in previous studies [13, 26, 27], and by genetic predisposition for MetS and Alzheimer's disease.

## Methods

### Study participants

The Epirus Health Study (EHS) is a deeply phenotyped ongoing population-based prospective cohort study. It was initiated in June 2019 and was designed to investigate the etiology of complex multifactorial chronic diseases in the Greek population. The EHS cohort consists of permanent residents of Epirus, a northwest geographical region in Greece, aged 21–77 years. Details of EHS have been published elsewhere [28].

**Fig. 1:**
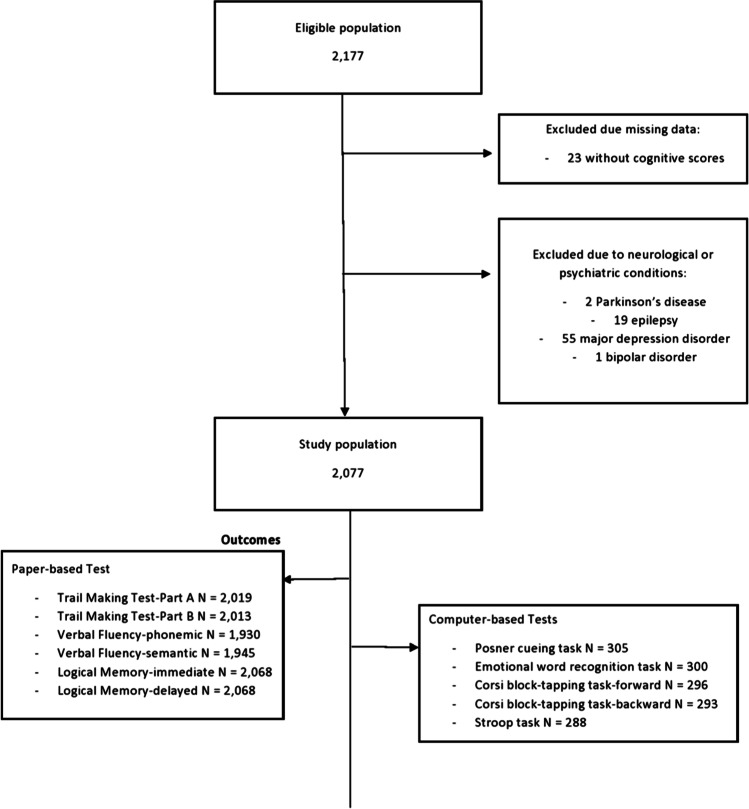
Flowchart of study participants

Until 30^th^ September 2022, a total of 2,177 participants were recruited. The analyses were performed on 2,077 participants after excluding 23 participants with missing data on cognitive scores and 77 participants who had self-reported serious neurological or psychiatric conditions at recruitment, namely 2 with Parkinson’s disease, 19 with epilepsy, 55 with major depression disorder and 1 with bipolar disorder (Figure 1).

Regarding the online neuropsychological examination, an invitation was sent via email on 28/9/2021 to all participants recruited until August 2021, and on the last day of each month to participants recruited ever since. Until 30^th^ September 2022, 305 participants had completed the computer-based neuropsychological tests, and secondary prospective analyses were performed on 155 participants that had completed the online neuropsychological examination at least 6 months after their initial neuropsychological examination at recruitment.

All participants provided written informed consent prior to participation in the study. The study was approved by the Research Ethics Committee of the University of Ioannina and is conducted in accordance with the Declaration of Helsinki.

### Data collection

A detailed data collection procedure has been presented previously [28]. Briefly, the EHS collects information on socio-demographic characteristics, lifestyle data, anthropometric, biochemical, clinical and cognitive measurements. At recruitment, participants underwent an interview and a clinical examination by two trained medical professionals. Basic demographic characteristics (i.e., age, sex, place of birth, marital status, level of education, current employment status and income), personal and family medical history, and lifestyle factors (i.e., physical activity, smoking habits, alcohol consumption) were acquired with a standard questionnaire. Weight, standing height and waist circumference were measured using SECA equipment. Systolic and diastolic blood pressures were measured using the MicroLife A6 PC-AFIB PC monitor. Βlood samples were collected after at least eight hours of overnight fast to measure serum glucose, total cholesterol, low- (LDL) and high- density (HDL) lipoprotein cholesterol and triglycerides.

### MetS definition

Presence of MetS was determined according to the NCEP-ATP III criteria revised by AHA/National Heart, Lung, and Blood Institute [7] that require three or more of the following conditions: waist circumference ≥102 cm in men or ≥88 cm in women, triglycerides ≥150 mg/dL or lipid lowering drug treatment, HDL cholesterol <40 mg/dL in men or <50 mg/dL in women or lipid lowering drug treatment, systolic blood pressure ≥130 mm Hg or diastolic blood pressure ≥85 mm Hg or anti-hypertensive drug treatment, fasting glucose ≥100 mg/dL or anti-diabetic drug treatment. We also used the IDF criteria as a sensitivity analysis to investigate the possible role of different exposure measurement on the results. IDF criteria require central obesity defined by ethnic-specific waist circumference (Europeans ≥94 cm in men or ≥80 in women) or body-mass index (BMI) over 30 kg/m^2^ and two of the following: triglycerides ≥150 mg/dL or drug treatment, HDL cholesterol <40 mg/dL in men or <50 mg/dL in women or drug treatment, systolic blood pressure ≥130 mm Hg or diastolic blood pressure ≥85 mm Hg or anti-hypertensive drug treatment, fasting glucose ≥100 mg/dL or previously diagnosed type 2 diabetes (Online Resource 1).

### Cognitive measurements

Cognitive functions were assessed using paper-based and computer-based neuropsychological tests. The paper-based Greek versions of the Trail Making Test (TMT) [29], the Verbal Fluency test (VF) [30] and the Logical Memory test (LM) [Kosmidis MH, Bozikas V, Vlahou CH, Giaglis G (2012) Unpulished Neuropsychological battery] [31], that have been standardized for age and education level, were administered at recruitment.

Computer-based neuropsychological tests were used for secondary prospective analyses and included the Posner cueing task [32], an emotional word recognition task [33], the Corsi block-tapping task [34] and the Stroop task [35]. Participants were invited to complete the computer-based neuropsychological tests through the well-established online platform PsyToolkit [36, 37].

In brief, TMT, Posner cueing task and Stroop task measure attention, VF and Stroop task measure executive functions and LM, the emotional word recognition task and Corsi block-tapping task measure memory. Total scores of VF, LM, the emotional word recognition task, and Corsi block-tapping task are the sum of correct responses and higher scores indicate better performance. Total score of TMT is the seconds needed to complete each subtest and thus, lower scores indicate better performance. Scores of Posner cueing task and Stroop task are both correct responses and seconds needed to complete each condition of test. Further details for each neuropsychological test and scoring are provided in the supplementary material (Online Resource 2).

### Genotyping and genetic risk score (GRS) calculation

Genotyping was performed using the Illumina Global Screening Array at the Erasmus Medical Center (Rotterdam, Netherlands). Imputation was performed using the Trans-Omics for Precision Medicine (TOPMed) program.

We selected the genetic variants to create the GRS for MetS from the most recent genome-wide association study (GWAS) conducted in an European sample [38]. Ninety-three single-nucleotide polymorphisms (SNPs) reaching genome-wide statistical significance (*p* < 5 × 10^–8^) were searched in the EHS database and seventy-six of them were found (Online Resource 3).

The genetic variants to create the GRS for Alzheimer’s disease (AD) was selected from a GWAS meta-analysis [39]. Twenty-nine SNPs reaching genome-wide statistical significance (*p* < 5 × 10^–8^) in the meta-analysis including all cohorts were included (Online Resource 4).

Assuming an additive genetic model, the GRS was calculated as the sum of the products after multiplying the number of the corresponding risk alleles (0, 1 or 2) for each SNP by the effect size (beta estimate) of that allele with disease risk in the aforementioned GWAS as implemented in PLINK software [40].

The genetic information was available for 1,143 participants in EHS. The analyses were performed in 1,099 participants with genotyping data after excluding participants with missing data on cognitive scores and with self-reported serious neurological or psychiatric conditions.

### Statistical analysis

Baseline characteristics of the study participants were summarized using means and standard deviations (SD) for continuous variables and percentages for categorical variables. Independent sample t-tests or x^2^ were applied to compare the baseline characteristics according to MetS status.

Multivariable linear regression models were employed to investigate the cross-sectional association of MetS status (absent vs present) and each individual component (absent vs present) with the cognitive abilities assessed by the continuous scores of the paper-based and computer-based neuropsychological tests.

All models were first adjusted for age (continuous), sex, and education (primary and secondary school, high school, higher education), and additionally adjusted for cardiovascular disease (absence or presence of stroke or ischemic heart disease or heart failure or other heart disease diagnosis), alcohol consumption (never, less than once/month, 1-3 times/month, 1-2 times/week, almost every day) and recreational physical activity [measured in Metabolic Equivalents of Task (MET) per hour/week] (continuous).

Interaction analyses of MetS status with sex, age groups (<60 years, ≥60 years) and with GRS for MetS and for AD were performed in the fully adjusted models. The GRS were standardized by subtracting the mean and then dividing by the standard deviation of the whole sample.

All statistical analyses were undertaken using STATA (version 14; StataCorp, College Station, TX, USA). The level of statistical significance was set at 0.05, but the interactions were interpreted more conservatively given the higher sample size needed for such analyses.

## Results

### Sociodemographic characteristics

Table 1 presents the sociodemographic characteristics of the study participants overall and by MetS status. Women (59.9%) and individuals of higher education (64.2%) preponderated in the sample. The mean age of study participants was 46.7 years (SD=11.4), approximately 42% of participants reported alcohol consumption of at least once per week and most of them had low to moderate physical activity level.

**Table 1:** Sociodemographic characteristics of study participants overall and by presence of metabolic syndrome (MetS).

Characteristics	All participants (*n*=2,077)	MetS based on NCEP-ATP III criteria		*p* value
		MetS (*n*=359)	No MetS (*n*=1,718)	
Age, years	46.66 ± 11.42	54.26 ± 9.65	45.08 ± 11.12	1.15e^-45 a^
Sex, Female	1,243 (59.85)	182 (50.70)	1,061 (61.76)	1.00e^-5 b^
Education				3.19e^-18 b^
Primary and secondary school*	159 (7.66)	67 (18.72)	92 (5.36)	
High school**	583 (28.10)	107 (29.89)	476 (27.72)	
Higher education***	1,333 (64.24)	184 (51.40)	1,149 (66.92)	
MetS components				
WC, cm	91.06 ± 14.32	105.26 ± 11.68	88.05 ± 12.96	2.78e^-106 a^
TG, mg/dL	96.09 ± 56.80	152.39 ± 82.89	84.11 ± 40.44	5.27e^-106 a^
HDL-C, mg/dL	54.45 ± 12.30	45.88 ± 10.94	56.28 ± 11.80	2.70e^-50 a^
On lipid lowering medication	344 (15.56)	160 (44.57)	184 (10.71)	1.64e^-55 b^
SBP, mm Hg	116.91 ± 13.25	127.37 ± 12.87	114.68 ± 12.23	3.07e^-65 a^
DBP, mm Hg	74.98 ± 11.42	83.11 ± 10.73	73.25 ± 10.80	1.50e^-52 a^
On antihypertensive medication	255 (12.28)	155 (43.18)	100 (5.82)	1.16e^-85 b^
FGlu, mg/dL	87.17 ± 15.05	98.23 ± 24.81	84.82 ± 10.61	5.36e^-56 a^
On antidiabetic medication	55 (2.65)	40 (11.14)	15 (0.87)	3.02e^-28 b^
Alcohol consumption				2.46e^-6 b^
Never	268 (12.90)	67 (18.66)	201 (11.70)	
Less than once/month	623 (30.00)	124 (34.54)	499 (29.05)	
1-3 times/month	314 (15.12)	30 (8.36)	284 (16.53)	
1-2 times/week	611 (29.42)	88 (24.51)	523 (30.44)	
Almost every day	261 (12.57)	50 (13.93)	211 (12.28)	
Physical activity, MET-hours/week	16.21 ± 23.18	9.36 ± 13.09	17.65 ± 24.54	6.27e^-10 a^
	All participants (*n*=1,099)	MetS (*n*=211)	No MetS (*n*=888)	*p* value
Genetic Risk Score for MetS	1.92e^-5^ ± 1.00	-0.037 ± 0.94	0.009 ± 1.01	0.55 ^a^
Genetic Risk Score for AD	3.47e^-7^ ± 1.00	0.021 ± 0.97	-0.005 ± 1.01	0.73 ^a^

*AD*, Alzheimer’s Disease; *DBP*, diastolic blood pressure; *FGlu*, fasting glucose; *HDL-C*, high-density lipoprotein cholesterol; *IDF*, International Diabetes Federation; *MET*, metabolic equivalents of energy expenditure; *NCEP-ATP III*, National Cholesterol Education/Adult Treatment Panel III; *SBP*, systolic blood pressure; *TG*, triglycerides; *WC*, waist circumference

*Elementary school or junior high school, up to 9 years of education. **High school, up to 12 years of education. ***University degree/MSc/PhD/Postdoc, more than 13 years of education

^a^Comparisons using t-test. ^b^ Comparisons using x^2^ test. Mean ± standard deviation and frequency (percentage) are presented for continuous and categorical variables, respectively

MetS according to the NCEP-ATP III criteria was identified in 359 (17.3%) study participants and according to the IDF criteria in 416 (20.3%) study participants. Of those with MetS according to NCEP-ATP III criteria, 66.02%, 27.86% and 6.13% met at least 3, 4 and all 5 criteria, respectively. Compared to those without MetS, MetS cases were older (mean difference=9.18, *p*=1.15e^-45^), and less physically active (mean diff=8.29, *p*=6.27e^-10^). MetS was also associated with sex (*p*=1.00e^-5^), education (*p*=3.19e^-18^) and alcohol consumption (*p*=2.46e^-6^). GRS did not differ between individuals with and without MetS.

### MetS and cognitive abilities

Mean cognitive scores and follow-up period for study participants overall and by MetS status are shown in Online Resource 5. Individuals with MetS had worse cognitive scores in all paper-based neuropsychological tests and worse cognitive scores in two computer-based neuropsychological tests (Emotional word recognition task, Stroop task). Follow-up period did not differ between individuals with and without MetS and ranged from 194 to 858 days (median 489 days) for all study participants.

In multivariable analyses, MetS was associated with poorer performance in tasks that assess attentional and memory abilities, but not for verbal fluency (Table 2). Individuals with MetS were on average 1.62 seconds slower in TMT-Part A and recalled an average of 0.62 fewer words in LM-immediate recall test than individuals without MetS. In the minimally adjusted multivariable linear regression models, individuals with MetS recalled significantly less words in LM-delayed recall test (β=-0.34 words, *p*=0.02) but the association became borderline significant (β=-0.29 words, *p*=0.06) in the fully adjusted models.

**Table 2:** Associations between presence of metabolic syndrome (MetS) and scores of paper-based neuropsychological tests (*N*=2,077).

Cognitive function scores	MetS based on NCEP-ATP III criteria			
	Model 1 ^a^		Model 2 ^b^	
	Beta	95% CI	Beta	95% CI
Trail Making Test				
Part A	1.66*	0.26, 3.07	1.62*	0.20, 3.04
Part B	0.68	-1.56, 2.93	0.61	-1.66, 2.88
Verbal Fluency				
Semantic	-0.10	-0.83, 0.62	0.09	-0.64, 0.82
Phonemic	-0.13	-0.58, 0.32	-0.03	-0.49, 0.42
Logical Memory				
Immediate recall	-0.70*	-1.26, -0.14	-0.62*	-1.19, -0.05
Delayed recall	-0.34*	-0.63, -0.05	-0.29	-0.58, 0.01

*CI*, confidence interval

*significant at *p*<0.05

^a^Adjusted for age (continuous), sex, education (primary and secondary school, high school, higher education). ^b^ Adjusted for age (continuous), sex, education (primary and secondary school, high school, higher education), cardiovascular disease (absence or presence of stroke or ischemic heart disease or heart failure or other heart disease diagnosis), alcohol consumption (never, less than once/month, 1-3 times/month, 1-2 times/week, almost every day) and physical activity (continuous)

Secondary prospective analyses that used computer-based tests replicated the association of MetS with lower scores in tasks that measure the cognitive domains of attention and memory but also executive functions (Table 3). Individuals with MetS made more intrusion errors in the emotional word recognition task (β=-2.07 words, *p*=0.04), a fact that implies executive dysfunction. Moreover, individuals with MetS had significantly worse performance in Stroop task (β=-7.07 correct answers, *p*=0.03), which measures processing speed and attention.

**Table 3:** Associations between presence of metabolic syndrome (MetS) and scores of computer-based neuropsychological tests (*N*=155).

Cognitive function scores	MetS based on NCEP-ATP III criteria			
	Model 1 ^a^		Model 2 ^b^	
	Beta	95% CI	Beta	95% CI
Posner cueing				
Total correct	-2.53	-7.75, 2.69	-1.48	-6.92, 3.97
Mean reaction time - valid trials	-0.02	-0.07, 0.04	0.00	-0.06, 0.06
Mean reaction time - invalid trials	-0.01	-0.07, 0.05	-0.01	-0.07, 0.05
Emotional word recognition				
Total correct	-1.91	-4.84, 1.03	-1.81	-4.85, 1.24
True positive	0.04	-1.57, 1.63	0.27	-1.36, 1.89
True negative	-1.94*	-3.87, -0.02	-2.07*	-4.09, -0.06
Corsi block-tapping				
Forward	-0.32	-1.20, 0.56	-0.23	-1.16, 0.69
Backward	0.24	-0.67, 1.15	0.20	-0.75, 1.16
Stroop				
Total correct	-7.50*	-13.58, -1.42	-7.07*	-13.31, -0.83
Mean reaction time - congruent trials	-0.06	-0.15, 0.03	-0.06	-0.15, 0.02
Mean reaction time - incongruent trials	0.01	-0.11, 0.12	0.00	-0.11, 0.12

*CI*, confidence interval

*significant at *p*<0.05

^a^Adjusted for age (continuous), sex, education (primary and secondary school, high school, higher education). ^b^ Adjusted for age (continuous), sex, education (primary and secondary school, high school, higher education), cardiovascular disease (absence or presence of stroke or ischemic heart disease or heart failure or other heart disease diagnosis), alcohol consumption (never, less than once/month, 1-3 times/month, 1-2 times/week, almost every day) and physical activity (continuous)

When examining the individual components of MetS, abdominal obesity was associated with lower performance in LM-delayed recall (β=-0.32 words, *p*=0.01), and hyperglycemia was associated with lower performance in LM-immediate recall (β=-0.76 words, *p*=0.04). Hypertension and high triglycerides were not associated with any cognitive function score, and the relationships of low HDL cholesterol with VF-semantic and LM-delayed recall were inverse but slightly attenuated in the fully adjusted models (Table 4).

**Table 4:** Association between presence of individual components of metabolic syndrome and scores of paper-based neuropsychological tests (*N*=2,077).

Cognitive function scores	Model 1 ^a^		Model 2 ^b^	
	Beta	95% CI	Beta	95% CI
Abdominal obesity				
Trail Making Test				
Part A	0.52	-0.56, 1.59	0.47	-0.62, 1.57
Part B	-0.48	-2.19, 1.24	-0.64	-2.38, 1.11
Verbal Fluency				
Semantic	-0.31	-0.86, 0.24	-0.16	-0.72, 0.40
Phonemic	-0.35*	-0.69, 0.01	-0.25	-0.60, 0.10
Logical Memory				
Immediate recall	-0.23	-0.66, 0.21	-0.14	-0.58, 0.31
Delayed recall	-0.37*	-0.59, -0.14	-0.32*	-0.55, -0.09
Elevated triglycerides				
Trail Making Test				
Part A	1.19	-0.05, 2.43	1.18	-0.06, 2.43
Part B	0.72	-1.27, 2.71	0.73	-1.27, 2.73
Verbal Fluency				
Semantic	-0.21	-0.86, 0.43	-0.10	-0.75, 0.54
Phonemic	0.05	-0.35, 0.45	0.09	-0.31, 0.49
Logical Memory				
Immediate recall	-0.47	-0.97, 0.03	-0.43	-0.93, 0.08
Delayed recall	-0.14	-0.40, 0.12	-0.11	-0.37, 0.15
Low HDL cholesterol				
Trail Making Test				
Part A	0.91	-0.19, 2.01	0.88	-0.23, 1.99
Part B	0.96	-0.79, 2.71	0.89	-0.88, 2.66
Verbal Fluency				
Semantic	-0.62*	-1.18, -0.06	-0.49	-1.06, 0.08
Phonemic	-0.17	-0.52, 0.18	-0.10	-0.45, 0.25
Logical Memory				
Immediate recall	-0.44	-0.88, 0.01	-0.37	-0.81, 0.08
Delayed recall	-0.26*	-0.49, -0.03	-0.23	-0.46, 0.00
High blood pressure				
Trail Making Test				
Part A	0.49	-0.72, 1.69	0.49	-0.72, 1.70
Part B	-0.39	-2.31, 1.54	-0.44	-2.36, 1.49
Verbal Fluency				
Semantic	-0.20	-0.82, 0.42	-0.12	-0.74, 0.50
Phonemic	-0.11	-0.49, 0.28	-0.06	-0.44, 0.33
Logical Memory				
Immediate recall	-0.16	-0.65, 0.32	-0.12	-0.61, 0.37
Delayed recall	-0.18	-0.43, 0.08	-0.15	-0.40, 0.10
Elevated fasting glucose				
Trail Making Test				
Part A	1.52	-0.25, 3.28	1.46	-0.32, 3.23
Part B	0.30	-2.52, 3.13	0.29	-2.55, 3.13
Verbal Fluency				
Semantic	0.76	-0.15, 1.67	0.92	-0.02, 1.83
Phonemic	0.10	-0.47, 0.66	0.17	-0.40, 0.74
Logical Memory				
Immediate recall	-0.80*	-1.51, -0.09	-0.76*	-1.47, -0.05
Delayed recall	-0.25	-0.62, 0.12	-0.21	-0.58, 0.16

*CI*, confidence interval

*significant at *p*<0.05

^a^Adjusted for age (continuous), sex, education (primary and secondary school, high school, higher education). ^b^ Adjusted for age (continuous), sex, education (primary and secondary school, high school, higher education), cardiovascular disease (absence or presence of stroke or ischemic heart disease or heart failure or other heart disease diagnosis), alcohol consumption (never, less than once/month, 1-3 times/month, 1-2 times/week, almost every day) and physical activity (continuous).

When the potential interactions by sex, age and GRS for MetS and AD were assessed, there was little evidence for interaction (Online Resource 6). An exception was the interaction of MetS with age for LM-delayed recall (p_interaction_=0.01), indicating a statistically significant lower performance in younger participants (β=-0.70 words, *p*=2.65e^-5^) that was not present in older participants (β=0.35, *p*=0.34). In addition, the interaction of MetS with GRS for MetS was evident for TMT-Part B, indicating a positive trend for participants with higher GRS for MetS, that was not present in participants with lower GRS for MetS.

Similar results were obtained when the IDF criteria were used for the definition of MetS (Online Resources 7–10). Some differences were noticed in tests assessing memory domain. Specifically, in LM-immediate recall (Online Resource 7), in emotional word recognition task (Online Resource 8), and in the association of obesity with LM-delayed recall (Online Resource 9), the directions were consistent with the main analyses using the NCEP-ATP III criteria, but the associations were no longer statistically significant.

## Discussion

In this study, the presence of MetS was cross-sectionally associated with lower performance mainly in tasks that assess attention and to a lesser extent in tasks that assess memory and executive functions. These associations were sustained in the secondary analyses using prospectively administered computer-based tests. Using the revised NCEP-ATP III criteria or the IDF criteria for the definition of MetS, did not have qualitative changes on the results.

Evidence of a possible association between MetS and attentional deficits has been found in some previous cross-sectional studies [15, 16, 41]. However, it should be stated that attention as a cognitive domain has not been commonly assessed. There is a larger number of recent cross-sectional studies that replicated the association between presence of MetS and memory impairments [42–47] as well as executive dysfunction [41, 42, 44, 46, 48]. In longitudinal studies, none of these domains were consistently associated with presence of MetS, but there are some studies that found significant association of MetS with decline in attention [49], memory [50, 51] or executive functions [51, 52].

Analyses of the individual components indicated that the association of MetS and worse performance on tasks of attention and memory in this study population could be driven by elevated fasting glucose and abdominal obesity. Elevated fasting glucose has been consistently associated with cognitive decline both individually [53–57] and as a component of MetS [15, 42, 46, 47, 58–61], even in the absence of significant association between cognitive decline and presence of MetS [19, 22, 62, 63]. Hyperglycemia, as a component of MetS, has been associated with decrements in various cognitive domains, including attention [15, 59, 63] and memory [46, 47, 59, 64], similar to our study finding of worse performance in LM-immediate recall. The suggested mechanistic pathways are reduced volume of frontal lobes regarding attentional abilities and reduced hippocampal volume regarding memory performance [65].

Abdominal obesity has been linked with adverse memory function through neuroinflammation in hippocampus caused by low-grade systemic inflammation that begins in adipose tissue and spreads into the brain [66, 67]. Nonetheless, studies that concluded a significant association of MetS and memory impairments reported more often hyperglycemia as a leading factor [46, 47].

Apart from the underlying mechanisms of individual components, brain changes associated with MetS as a whole could explain the findings of the present study. Although still not fully understood, it is proposed that the pathophysiology of MetS causes microvascular damage that results in white matter abnormalities [68], a condition that is related with processing speed/attention deficits in general [69] and in individuals with metabolic syndrome particularly [70, 71].

No interaction between presence of MetS and sex was found, in line with previous cross-sectional studies [17, 72]. However, the existence of studies showing a female [26, 64] or a male disadvantage [27, 73] indicates that the sex-related biological mechanisms remain largely unknown. The significant interaction observed between presence of MetS and age groups is in accordance with an earlier cross-sectional study [13] that found steeper decline associated with MetS in a verbal learning test only for middle-aged but not for older participants. The null association of presence of MetS on cognition in older people has been also found in longitudinal studies [74, 75], and has been attributed to survival bias. Nonetheless, in our study there were not enough participants aged over 65 years and consequently the old group was defined using the cut-off of 60 years instead of at least 65 years, as used in the aforementioned studies. Thus, this finding should be interpreted with caution. The significant interaction between presence of MetS and GRS for MetS with TMT-Part B indicates that the executive dysfunction is probably more evident in people that except for the phenotype, they have also stronger genetic predisposition for this particular phenotype. However, this association needs replication in future studies.

This study has several strengths. We recruited a large population-based sample and we used standardized methodology for exposure and outcome measurements. Presence of MetS was defined by the two most widely employed criteria, revised NCEP-ATP III and IDF, allowing comparisons for MetS validity according to different criteria. Assessment of cognitive function was accomplished using widely administered neuropsychological procedures, and in the secondary analyses using prospectively administered computer-based tests to assure more accurate measurements. Furthermore, a novelty of this study is the investigation of possible genetic contributions, as expressed by the utilization of GRS for MetS and AD, to the association between presence of MetS and cognitive function [26].

An important limitation is the cross-sectional design of the primary analysis which precludes the inference of causality. We did however use a prospective additional investigation with computer-based neuropsychological tests but only 14.6% of the population completed them. Also, a limited number of traits for MetS definition (i.e., diabetes diagnosis, medication intake) was based on self-reporting, and despite the use of standardized neuropsychological procedures, not all cognitive domains were examined. Finally, given the relatively small sample and the non-independence of the examined exposures and outcomes, we chose not to control for multiple testing.

In conclusion, the results of our study are supportive of the hypothesis that MetS is associated with vascular cognitive impairment, a cognitive profile with predominant deficits in processing speed, attention and to a smaller degree in memory. Future research is needed with neuroimaging data and more extensive cognitive testing to clarify the association between presence of MetS and cognitive function, and to elucidate the potential mediation effects of brain structures and networks.

## Supplementary information

ESM 1 Online Resource 1. Diagnostic criteria for the definition of metabolic syndrome. (PDF 435 kb)

ESM 2 Online Resource 2. Detailed description of neuropsychological tests used. (PDF 214 kb)

ESM 3 Online Resource 3. Genotypic information for the single nucleotide polymorphisms included in the calculation of genetic risk score for Metabolic Syndrome. (PDF 519 kb)

ESM 4 Online Resource 4. Genotypic information for the single nucleotide polymorphisms included in the calculation of genetic risk score for Alzheimer’s Disease. (PDF 438 kb)

ESM 5 Online Resource 5. Cognitive scores and follow-up period of study participants overall and by presence of metabolic syndrome (MetS). (PDF 642 kb)

ESM 6 Online Resource 6. Interaction analysis of metabolic syndrome (MetS) based on National Cholesterol Education-Adult Treatment Panel III (NCEP-ATP III) criteria and paper-based neuropsychological tests by (A) sex, (B) age, (C) Genetic Risk Score for MetS and (D) Genetic Risk Score for Alzheimer’s Disease. (PDF 423 kb)

ESM 7 Online Resource 7. Associations between presence of metabolic syndrome (MetS) based on International Diabetes Federation (IDF) criteria and scores of paper-based neuropsychological tests (N=2,077). (PDF 442 kb)

ESM 8 Online Resource 8. Associations between presence of metabolic syndrome (MetS) based on International Diabetes Federation (IDF) criteria and scores of computer-based neuropsychological tests (N=155). (PDF 454 kb)

ESM 9 Online Resource 9. Associations between presence of individual components of metabolic syndrome based on International Diabetes Federation (IDF) criteria and scores of paper-based neuropsychological tests (N=2,077). (PDF 705 kb)

ESM 10 Online Resource 10. Interaction analysis of metabolic syndrome (MetS) based on International Diabetes Federation (IDF) criteria and paper-based neuropsychological tests by (A) sex, (B) age, (C) Genetic Risk Score for MetS and (D) Genetic Risk Score for Alzheimer’s Disease. (PDF 404 kb)

## Data Availability

The datasets generated during during the current study are available from the corresponding author on reasonable request.
