# Supplementary material for: Metabolic syndrome and cognitive deficits in the Greek cohort of Epirus Health Study
Source: Neurol Sci. 2023 May 10;44(10):3523–33. doi: 10.1007/s10072-023-06835-4 (PMC10495510; doi:10.1007/s10072-023-06835-4)
Supplement: Supplementary file 1 — Online Resource 1. Diagnostic criteria for the definition of metabolic syndrome. (PDF 435 kb) [file 10072_2023_6835_MOESM1_ESM.pdf]

Metabolic syndrome and cognitive deficits in the Greek cohort of Epirus Health Study, Neurological Sciences, Koutsonida M, Koskeridis F, Markozannes G, Kanellopoulou A, Mousas A, Ntotsikas E, Ioannidis P, Aretouli E and Tsilidis KK; Department of Epidemiology and Biostatistics, School of Public Health, Imperial College London, London, United Kingdom, k.tsilidis@imperial.ac.uk (KKT)

Supplementary Table 1. Diagnostic criteria for the definition of metabolic syndrome.

	**Revised NCEP-ATP III (2005)**<br>**≥3 of the following**	**IDF (2005)**<br>**BMI/Central obesity + ≥2 of the following**
**Central obesity**	WC ≥ 102 cm (men)<br>WC ≥ 88 cm (women)	WC (ethnicity-specific values)<br>For Europeans:<br>WC ≥ 94 cm (men)<br>WC ≥ 80 cm (women)
**BMI**		> 30 kg/m^2
**Triglycerides**	≥150 mg/dL<br>OR Lipid lowering medication	≥ 150 mg/dL<br>OR Lipid lowering medication
**HDL-C**	< 40 mg/dL (men)<br>< 50 mg/dL (women)<br>OR Lipid lowering medication	< 40 mg/dL (men)<br>< 50 mg/dL (women)<br>OR Lipid lowering medication
**Blood pressure**	SBP ≥ 130 mmHg<br>OR DBP ≥ 85 mmHg<br>OR Antihypertensive medication	SBP ≥ 130 mmHg<br>OR DBP ≥ 85 mmHg<br>OR Antihypertensive medication
**Fasting glucose**	≥100 mg/dL<br>OR Antidiabetic medication	≥ 100 mg/dL
**Diabetes**		Type 2 Diabetes

Abbreviations: BMI, Body mass index; DBP, diastolic blood pressure; HDL-C, high-density lipoprotein cholesterol; IDF, International Diabetes Federation; NCEP-ATP III, National Cholesterol Education-Adult Treatment Panel III; SBP, systolic blood pressure; WC, waist circumference.
