# Supplementary material for: Metabolic syndrome and cognitive deficits in the Greek cohort of Epirus Health Study
Source: Neurol Sci. 2023 May 10;44(10):3523–33. doi: 10.1007/s10072-023-06835-4 (PMC10495510; doi:10.1007/s10072-023-06835-4)
Supplement: Supplementary file 2 — Online Resource 2. Detailed description of neuropsychological tests used. (PDF 214 kb) [file 10072_2023_6835_MOESM2_ESM.pdf]

Metabolic syndrome and cognitive deficits in the Greek cohort of Epirus Health Study, Neurological Sciences, Koutsonida M, Koskeridis F, Markozannes G, Kanellopoulou A, Mousas A, Ntotsikas E, Ioannidis P, Aretouli E and Tsilidis KK; Department of Epidemiology and Biostatistics, School of Public Health, Imperial College London, London, United Kingdom, k.tsilidis@imperial.ac.uk (KKT)

Online Resource 2. Detailed description of neuropsychological tests used.

The Trail Making Test (TMT) is a test of attention and executive cognition. Participants were instructed to connect a set of dots with numbers in an ascending order as quickly as possible while maintaining accuracy (Trail Making-Part A – TMT-A), and then to connect a set of dots with numbers and letters in an ascending pattern with the added task of switching between the numbers and letters (Trail Making-Part B – TMT-B). Scoring was based on seconds needed to complete the test with lower scores indicating better cognitive abilities.

The Verbal Fluency test (VF) is a measure of executive cognition that relies also on a language component. Participants were asked to generate as many words as possible in 60 seconds from the semantic category "animals" (semantic fluency subtest) and words beginning with the Greek letter "X" (phonemic fluency subtest). The total score was the sum of the words produced in each subtest with higher scores indicating better cognitive abilities.

The Logical Memory test (LM) is a verbal episodic memory test. Participants were read a story and asked to recite the story from memory immediately after its presentation twice (immediate recall condition) and approximately 20 minutes later (delayed recall condition). The number of correctly recalled information compiled the total score, ranging from 0-32 for the immediate recall condition and 0-16 for the delayed recall condition. Higher scores indicate better memory skills.

The Posner cueing task is a test of visual attention. Participants were instructed to fixate at a cross mark that was presented in the center of the computer screen and press a key to respond as quickly as possible when a target appeared on the left or on the right of the fixation point. The location of the target (valid trial) or the opposite location (invalid trial) may have been cued before target presentation. Total correct responses (0-50) and mean reaction time measured in seconds for correct valid trials and invalid trials were calculated separately. Higher scores in total correct responses but lower scores in mean time scores indicate better cognitive abilities.

Emotional word recognition task has been developed as a visual emotional memory recognition test. It consists of 72 words (36 targets and 36 distractors) selected from the Greek version of the Affective Norms for English Words Lexicon (Palogiannidi, Koutsakis, Iosif, & Potamianos, 2016). Stimuli were chosen based on their valence (24 positive, 24 negative or 24 neutral). In the study phase each word of the 36 targets was presented sequentially for 3.3 seconds in the center of the screen, and after the completion of the study phase, participants were presented sequentially the 72 words (36 targets and 36 distractors) and had to respond in 15 seconds if they remember the word or not. Total recognition score (0-72), True Positive score (0-36) and True Negative score (0-36) were calculated. Higher scores indicate better cognitive abilities.

The Corsi block-tapping task is a measure of visuo-spatial working memory. Nine square frames were impartially arranged on the screen and flashed in a specific ascending sequence. At the end of the sequence presentation, a warning sound signal was informing participants to reproduce the sequence by clicking on the squares that flashed in the same (forward condition) or reversed order (backward condition). Task terminated when participants failed to reproduce two trials of the same

sequence length. The spans for forward and backward condition were derived. Higher number of the span indicated better cognitive abilities.

The Stroop task assesses attention and executive functions. Words of four color names (green, blue, red, yellow) were sequentially displayed in the center of the screen with their font color being same (congruent trial) or different (incongruent trial) as the semantic meaning of the word. Participants were instructed to identify as quickly as possible the font color of the presented words using specified keyboard arrows that corresponded to the four colors. Total correct responses (0-50) and mean reaction time for correct congruent trials and incongruent trials were calculated separately, as well as the Stroop effect as calculated by subtracting mean reaction time measured in seconds for correct incongruent trials from the mean reaction time for correct congruent trials. Higher scores in total correct responses but lower scores in mean time scores indicate better cognitive abilities.
