# Supplementary material for: Metabolic syndrome and cognitive deficits in the Greek cohort of Epirus Health Study
Source: Neurol Sci. 2023 May 10;44(10):3523–33. doi: 10.1007/s10072-023-06835-4 (PMC10495510; doi:10.1007/s10072-023-06835-4)
Supplement: Supplementary file 3 — Online Resource 3. Genotypic information for the single nucleotide polymorphisms included in the calculation of genetic risk score for Metabolic Syndrome. (PDF 519 kb) [file 10072_2023_6835_MOESM3_ESM.pdf]

Metabolic syndrome and cognitive deficits in the Greek cohort of Epirus Health Study, Neurological Sciences, Koutsonida M, Koskeridis F, Markozannes G, Kanellopoulou A, Mousas A, Ntotsikas E, Ioannidis P, Aretouli E and Tsilidis KK; Department of Epidemiology and Biostatistics, School of Public Health, Imperial College London, London, United Kingdom, k.tsilidis@imperial.ac.uk (KKT)

Online Resource 3. Genotypic information for the single nucleotide polymorphisms (SNPs) included in the calculation of genetic risk score for Metabolic Syndrome.

Chr	Position	Gene	SNP	Effect allele	beta	SE
1	26695422	ARID1A	rs114165349	C	0.147253	0.130037
1	39582337	PABPC4, HEYL	rs11206374	A	0.065077	0.105334
1	62440818	USP1	rs638714	T	0.051495	0.118824
1	93371576	Y_RNA, DR1	rs12752223	T	0.037483	0.15711
1	177944384	SEC16B, CRYZL2P-SEC16B	rs10913469	C	0.048264	0.143977
1	230161390	GALNT2	rs2281721	C	0.082075	0.074616
2	422144	LINC01865, LINC01874	rs62107261	C	0.089776	0.148048
2	620297	LINC01875, TMEM18	rs7563362	A	0.061357	0.135369
2	21014672	APOB	rs673548	A	0.09779	0.078254
2	27508073	GCKR	rs1260326	T	0.053697	0.112925
2	65048915	LINC02245, LINC02576	rs1009360	C	0.043589	0.127834
2	164675944	COBLL1	rs10187501	G	0.046559	0.129399
2	170772553	Y_RNA, EIF2S2P4	rs12472667	G	0.03749	0.157023
2	226230443	MIR5702, NYAP2	rs2138161	T	0.075241	0.085682
3	136236137	PCCB, MSL2	rs61789601	T	0.068372	0.105029
3	157079859	LINC00880, LEKR1	rs10049088	T	0.044322	0.137229
4	36075982	ARAP2	rs73123462	T	0.149549	0.148313
4	87061724	AFF1	rs7660883	G	0.044388	0.136469
4	102267552	SLC39A8	rs13107325	T	0.07609	0.139412
5	75707853	POC5	rs2307111	C	0.036672	0.158175
6	31358512	HLA-B, DHFRP2	rs9378248	A	0.044068	0.140153
6	32610856	HLA-DRB1, HLA-DQA1	rs5021727	G	0.037134	0.151786
6	34205553	CYCSP55, KRT18P9	rs76376137	G	0.082033	0.154291
6	34609480	ILRUN	rs11754773	G	0.089294	0.111018
6	43790159	VEGFA, LINC02537	rs998584	A	0.070685	0.088051
6	127118902	RSPO3	rs577721086	C	0.090094	0.141976
6	139512875	ATP5PBP6, LINC01625	rs632057	T	0.048679	0.123411
6	160671406	PLG, LPA	rs11751347	T	0.062488	0.144834

6	162553872	PRKN	rs10945840	C	0.038293	0.156109
7	26357619	SNX10	rs1534696	C	0.042436	0.132312
7	73464315	BAZ1B	rs12056034	G	0.096837	0.096505
7	130746210	KLF14, H4P1	rs10260148	T	0.052129	0.120377
7	150960007	KCNH2	rs56282717	A	0.046096	0.142928
8	9325848	RNU6-526P, RNU6-1151P	rs9987289	A	0.087388	0.112748
8	18414867	NAT2, PSD3	rs4921913	C	0.042644	0.156915
8	20003850	LPL	rs3844510	C	0.191134	0.048027
8	20079176	RPL30P9	rs28597716	G	0.152184	0.056484
8	31006422	PURG	rs10954772	T	0.039198	0.153906
8	115551448	TRPS1	rs3808439	A	0.038346	0.145502
8	125495066	LINC00861, TRIB1	rs2980888	T	0.095952	0.069709
9	89563557	SEMA4D, GADD45G	rs3949781	T	0.03802	0.149855
9	104884738	ABCA1	rs11789603	T	0.058891	0.156295
10	21590155	MLLT10	rs9971210	G	0.03658	0.149137
10	63311455	JMJD1C	rs10822155	A	0.051119	0.114662
10	98012647	CRTAC1	rs563296	G	0.04194	0.138314
11	27701787	BDNF	rs56133711	A	0.04433	0.145853
11	47508395	CELF1	rs7124681	A	0.055786	0.108461
11	61830500	FADS2	rs1535	G	0.079213	0.08187
11	65061370	NAALADL1, CDCA5	rs35661464	T	0.04638	0.1398
11	65638129	SIPA1	rs2306363	T	0.048276	0.14385
11	116778201	ZPR1	rs964184	G	0.252628	0.033931
11	118494495	KMT2A	rs9332817	C	0.115795	0.155319
12	49853685	BCDIN3D, RPL35AP28	rs7138803	A	0.036957	0.154213
12	122703928	HCAR3, HCAR2	rs56959712	T	0.047049	0.148955
15	41563538	TYRO3	rs1023193	T	0.045397	0.13372
15	43735687	CATSPER2P1	rs139974673	C	0.160596	0.111952
15	58386521	ALDH1A2	rs261290	T	0.077956	0.08451
16	15054789	PDXDC1, NTAN1	rs11075253	A	0.046879	0.134341
16	24715743	TNRC6A	rs7188873	A	0.03863	0.15164
16	29983601	TAOK2	rs3814883	T	0.039392	0.141677
16	53772541	FTO	rs56094641	G	0.073677	0.084808
16	56956804	CETP, HERPUD1	rs247617	A	0.196432	0.034077
16	81501185	CMIP	rs2925979	T	0.06161	0.100342
17	7581888	CD68	rs1143015	A	0.04914	0.158172
17	42561053	COASY, HSD17B1	rs12945575	T	0.044779	0.149288

17	43848758	CD300LG	rs72836561	T	0.23953	0.061094
17	49286745	FLJ40194	rs11655056	C	0.035993	0.157397
17	67844693	BPTF	rs11871285	T	0.047341	0.145796
18	23540071	NPC1	rs7239575	C	0.043234	0.132002
18	49621376	SMUG1P1, LIPG	rs1105654	G	0.038799	0.149413
18	60181418	MC4R, RNU4-17P	rs66922415	G	0.067846	0.102856
19	8364439	ANGPTL4	rs116843064	A	0.337476	0.073802
19	44912921	APOC1, APOE	rs483082	T	0.08993	0.079046
19	47068681	ZC3H4	rs1532127	G	0.04721	0.130752
20	44413724	HNF4A	rs1800961	T	0.138848	0.103275
20	64080700	OPRL1	rs8121509	C	0.036069	0.156318

Abbreviations: Chr, chromosome; SE, standard error.
