# Supplementary material for: Metabolic syndrome and cognitive deficits in the Greek cohort of Epirus Health Study
Source: Neurol Sci. 2023 May 10;44(10):3523–33. doi: 10.1007/s10072-023-06835-4 (PMC10495510; doi:10.1007/s10072-023-06835-4)
Supplement: Supplementary file 4 — Online Resource 4. Genotypic information for the single nucleotide polymorphisms included in the calculation of genetic risk score for Alzheimer’s Disease. (PDF 438 kb) [file 10072_2023_6835_MOESM4_ESM.pdf]

Metabolic syndrome and cognitive deficits in the Greek cohort of Epirus Health Study, Neurological Sciences, Koutsonida M, Koskeridis F, Markozannes G, Kanellopoulou A, Mousas A, Ntotsikas E, Ioannidis P, Aretouli E and Tsilidis KK; Department of Epidemiology and Biostatistics, School of Public Health, Imperial College London, London, United Kingdom, k.tsilidis@imperial.ac.uk (KKT)

Online Resource 4. Genotypic information for the single nucleotide polymorphisms (SNPs) included in the calculation of genetic risk score for Alzheimer's disease.

Chr	Position	Gene	SNP	Effect allele	beta	SE
1	161155392	ADAMTS4	rs4575098	A	0.016412	0.002577
1	207786828	CR1	rs2093760	A	0.02434	0.002742
2	127891427	BIN1	rs4663105	C	0.031095	0.00222
2	233981912	INPPD5	rs10933431	G	-0.01544	0.002509
3	57226150	HESX1	rs184384746	T	0.200409	0.035193
4	11026028	CLNK	rs6448453	A	0.014705	0.002451
6	32583357	HLA-DRB1	rs6931277	T	-0.01934	0.002969
6	40942196	TREM2	rs187370608	A	0.233272	0.02824
6	47432637	CD2AP	rs9381563	C	0.014451	0.002272
7	99971834	ZCWPW1	rs1859788	A	-0.0184	0.002313
7	143108158	EPHA1	rs7810606	T	-0.01452	0.002183
7	145950029	CNTNAP2	rs114360492	T	0.178834	0.029855
8	27464929	CLU/PTK2B	rs4236673	A	-0.02016	0.002229
10	11717397	ECHDC3	rs11257238	C	0.012943	0.002261
11	59958380	MS4A6A	rs2081545	A	-0.01787	0.00223
11	85776544	PICALM	rs867611	G	-0.02043	0.002324
11	121435587	SORL1	rs11218343	C	-0.03593	0.005255
14	92938855	SLC24A4	rs12590654	A	-0.01483	0.002308
15	59022615	ADAM10	rs442495	C	-0.01372	0.002258
15	63569902	APH1B	rs117618017	T	0.018027	0.003267
16	31133100	KAT8	rs59735493	A	-0.01299	0.00236
17	5138980	SCIMP	rs113260531	A	0.019986	0.003251
17	47450775	ABI3	rs28394864	A	0.012302	0.00218
18	56189459	ALPK2	rs76726049	C	0.056797	0.010288
19	1039323	ABCA7	rs111278892	G	0.019912	0.00305
19	45351516	APOE	rs41289512	G	0.206303	0.005785
19	46241841	AC074212.3	rs76320948	T	0.034933	0.006366
19	51727962	CD33	rs3865444	A	-0.01376	0.002355

20	54998544	CASS4	rs6014724	G	-0.02289	0.003688

Abbreviations: Chr, chromosome; SE, standard error.
