# Supplementary material for: Metabolic syndrome and cognitive deficits in the Greek cohort of Epirus Health Study
Source: Neurol Sci. 2023 May 10;44(10):3523–33. doi: 10.1007/s10072-023-06835-4 (PMC10495510; doi:10.1007/s10072-023-06835-4)
Supplement: Supplementary file 5 — Online Resource 5. Cognitive scores and follow-up period of study participants overall and by presence of metabolic syndrome (MetS). (PDF 642 kb) [file 10072_2023_6835_MOESM5_ESM.pdf]

Metabolic syndrome and cognitive deficits in the Greek cohort of Epirus Health Study, Neurological Sciences, Koutsonida M, Koskeridis F, Markozannes G, Kanellopoulou A, Mousas A, Ntotsikas E, Ioannidis P, Aretouli E and Tsilidis KK; Department of Epidemiology and Biostatistics, School of Public Health, Imperial College London, London, United Kingdom, k.tsilidis@imperial.ac.uk (KKT)

Online Resource 5. Cognitive scores and follow-up period of study participants overall and by presence of metabolic syndrome (MetS).

**Cognitive function scores**	**All participants (n=2,077)**	**MetS based on NCEP-ATP III criteria**		**p value**
		**MetS (n=359)**	**No MetS (n=1,718)**	
**Paper-based tests**				
Trail Making Test				
Part A	35.34 ± 12.58	39.93 ± 11.42	34.36 ± 11.42	$3.06e^{-14}$
Part B	65.79 ± 19.94	71.62 ± 22.48	64.57 ± 19.15	$1.60e^{-9}$
Verbal Fluency				
Semantic	21.92 ± 6.00	20.86 ± 6.46	22.13 ± 5.89	$5.00e^{-4}$
Phonemic	10.04 ± 3.74	9.22 ± 3.68	10.21 ± 3.73	$1.50e^{-5}$
Logical Memory				
Immediate recall	23.18 ± 4.89	21.62 ± 4.96	23.51 ± 4.82	$2.41e^{-11}$
Delayed recall	13.40 ± 2.55	12.59 ± 2.69	13.57 ± 2.49	$2.35e^{-11}$
	**All participants (n=156)**	**MetS (n=25)**	**No MetS (n=131)**	**p value**
**Follow-up period**, days	289.25 ± 274.60	334.82 ± 257.10	282.59 ± 276.90	0.27
**Computer-based tests**				
Posner cueing				
Total correct	43.73 ± 11.34	41.96 ± 11.63	44.07 ± 11.30	0.40
Mean reaction time - valid trials	0.49 ± 0.12	0.49 ± 0.13	0.49 ± 0.12	0.95
Mean reaction time - invalid trials	0.62 ± 0.14	0.63 ± 0.11	0.62 ± 0.14	0.56
Emotional word recognition				
Total correct	58.17 ± 6.63	55.44 ± 6.04	58.70 ± 6.63	0.02
True positive	30.71 ± 3.82	30.20 ± 3.82	30.81 ± 3.52	0.43
True negative	27.46 ± 4.33	25.24 ± 4.02	27.89 ± 4.27	$4.70e^{-3}$
Corsi block-tapping				
Forward	5.08 ± 1.92	4.80 ± 2.22	5.13 ± 1.86	0.43
Backward	5.03 ± 1.95	5.04 ± 1.49	5.02 ± 2.03	0.97

Stroop				
Total correct	35.73 ± 13.98	26.52 ± 13.73	37.46 ± 13.40	$5.00e^{-4}$
Mean reaction time - congruent trials	1.02 ± 0.21	1.04 ± 0.18	1.02 ± 0.22	0.68
Mean reaction time - incongruent trials	1.12 ± 0.25	1.17 ± 0.22	1.10 ± 0.25	0.24

Mean ± standard deviation are presented.
