# Supplementary material for: Metabolic syndrome and cognitive deficits in the Greek cohort of Epirus Health Study
Source: Neurol Sci. 2023 May 10;44(10):3523–33. doi: 10.1007/s10072-023-06835-4 (PMC10495510; doi:10.1007/s10072-023-06835-4)
Supplement: Supplementary file 6 — Online Resource 6. Interaction analysis of metabolic syndrome (MetS) based on National Cholesterol Education-Adult Treatment Panel III (NCEP-ATP III) criteria and paper-based neuropsychological tests by (A) sex, (B) age, (C) Genetic Risk Score for MetS and (D) Genetic Risk Score for Alzheimer’s Disease. (PDF 423 kb) [file 10072_2023_6835_MOESM6_ESM.pdf]

Metabolic syndrome and cognitive deficits in the Greek cohort of Epirus Health Study, Neurological Sciences, Koutsonida M, Koskeridis F, Markozannes G, Kanellopoulou A, Mousas A, Ntotsikas E, Ioannidis P, Aretouli E and Tsilidis KK; Department of Epidemiology and Biostatistics, School of Public Health, Imperial College London, London, United Kingdom, k.tsilidis@imperial.ac.uk (KKT)

Online Resource 6. Interaction analysis of metabolic syndrome (MetS) based on National Cholesterol Education-Adult Treatment Panel III (NCEP-ATP III) criteria and paper-based neuropsychological tests by (A) sex, (B) age, (C) Genetic Risk Score for MetS and (D) Genetic Risk Score for Alzheimer's Disease.

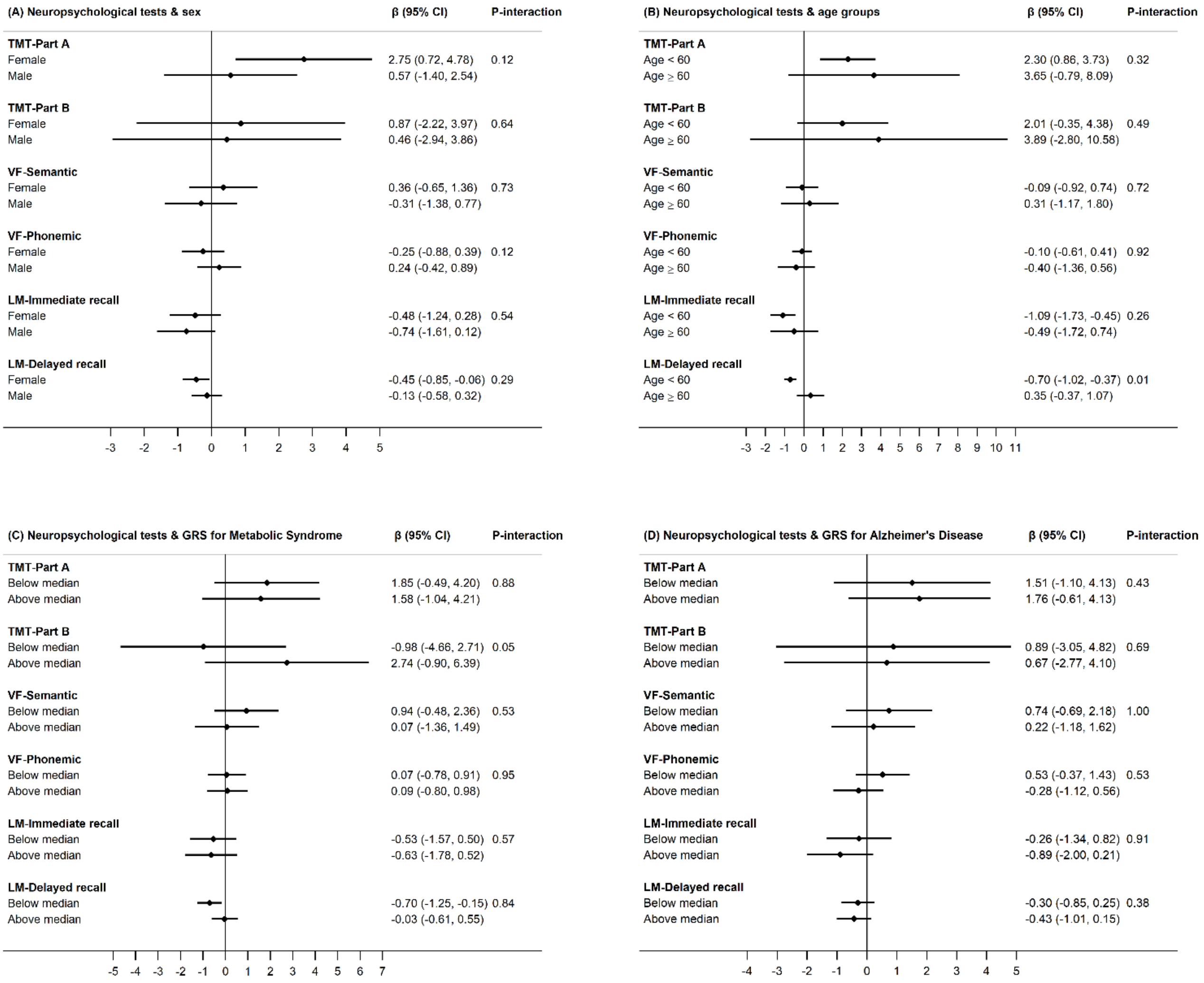

Abbreviations: GRS, Genetic Risk Score; LM, Logical memory; TMT, Trail Making Test; VF, Verbal fluency.

Footnote: The analyses were run using the continuous GRS for MetS and for Alzheimer's Disease but they were dichotomized at the median for illustrative reasons.
