# Supplementary material for: Metabolic syndrome and cognitive deficits in the Greek cohort of Epirus Health Study
Source: Neurol Sci. 2023 May 10;44(10):3523–33. doi: 10.1007/s10072-023-06835-4 (PMC10495510; doi:10.1007/s10072-023-06835-4)
Supplement: Supplementary file 8 — Online Resource 8. Associations between presence of metabolic syndrome (MetS) based on International Diabetes Federation (IDF) criteria and scores of computer-based neuropsychological tests (N=155). (PDF 454 kb) [file 10072_2023_6835_MOESM8_ESM.pdf]

Metabolic syndrome and cognitive deficits in the Greek cohort of Epirus Health Study, Neurological Sciences, Koutsonida M, Koskeridis F, Markozannes G, Kanellopoulou A, Mousas A, Ntotsikas E, Ioannidis P, Aretouli E and Tsilidis KK; Department of Epidemiology and Biostatistics, School of Public Health, Imperial College London, London, United Kingdom, k.tsilidis@imperial.ac.uk (KKT)

Online Resource 8. Associations between presence of metabolic syndrome (MetS) based on International Diabetes Federation (IDF) criteria and scores of computer-based neuropsychological tests (N=155).

Cognitive function scores	MetS based on IDF criteria			
	Model 1 [a]		Model 2 [b]	
	Beta	95% CI	Beta	95% CI
**Posner cueing**				
Total correct	-1.49	-6.22, 3.23	-0.40	-5.25, 4.45
Mean reaction time - valid trials	0.00	-0.06, 0.05	0.01	-0.05, 0.07
Mean reaction time - invalid trials	-0.01	-0.07, 0.05	0.00	-0.06, 0.06
**Emotional word recognition**				
Total correct	-1.05	-3.87, 1.76	-0.96	-3.85, 1.93
True positive	0.43	-1.10, 1.96	0.66	-0.86, 2.19
True negative	-1.48	-3.33, 0.37	-1.63	-3.53, 0.28
**Corsi block-tapping**				
Forward	-0.10	-0.94, 0.74	-0.01	-0.88, 0.86
Backward	-0.12	-0.99, 0.75	-0.15	-1.05, 0.75
**Stroop**				
Total correct	-8.74*	-14.45, -3.03	-8.37*	-14.17, -2.57
Mean reaction time - congruent trials	-0.02	-0.11, 0.06	-0.03	-0.11, 0.06
Mean reaction time - incongruent trials	0.04	-0.07, 0.14	0.04	-0.07, 0.15

Abbreviation: CI, confidence interval.

* significant at p<0.05

[a] Adjusted for age (continuous), sex, education (primary and secondary school, high school, higher education), cardiovascular disease (absence or presence of stroke or ischemic heart disease or heart failure or other heart disease diagnosis), alcohol consumption (never, less than once/month, 1-3 times/month, 1-2 times/week, almost every day), physical activity (continuous) and follow-up period (continuous). [b] Adjusted for age (continuous), sex, education (primary and secondary school, high school, higher education), cardiovascular disease (absence or presence of stroke or ischemic heart disease or heart failure or other heart disease diagnosis), alcohol consumption (never, less than once/month, 1-3 times/month, 1-2 times/week, almost every day) and physical activity (continuous).
