# Supplementary material for: Metabolic syndrome and cognitive deficits in the Greek cohort of Epirus Health Study
Source: Neurol Sci. 2023 May 10;44(10):3523–33. doi: 10.1007/s10072-023-06835-4 (PMC10495510; doi:10.1007/s10072-023-06835-4)
Supplement: Supplementary file 9 — Online Resource 9. Associations between presence of individual components of metabolic syndrome based on International Diabetes Federation (IDF) criteria and scores of paper-based neuropsychological tests (N=2,077). (PDF 705 kb) [file 10072_2023_6835_MOESM9_ESM.pdf]

Metabolic syndrome and cognitive deficits in the Greek cohort of Epirus Health Study, Neurological Sciences, Koutsonida M, Koskeridis F, Markozannes G, Kanellopoulou A, Mousas A, Ntotsikas E, Ioannidis P, Aretouli E and Tsilidis KK; Department of Epidemiology and Biostatistics, School of Public Health, Imperial College London, London, United Kingdom, k.tsilidis@imperial.ac.uk (KKT)

Online Resource 9. Associations between presence of individual components of metabolic syndrome based on International Diabetes Federation (IDF) criteria and scores of paper-based neuropsychological tests (N=2,077).

	**Model 1 [a]**		**Model 2 [b]**	
	**Beta**	**95% CI**	**Beta**	**95% CI**
**Abdominal obesity**				
Trail Making Test				
Part A	0.420	-0.70, 1.54	0.462	-0.67, 1.59
Part B	-0.808	-2.59, 0.97	-0.824	-2.63, 0.98
Verbal Fluency				
Semantic	-0.120	-0.69, 0.45	0.053	-0.52, 0.63
Phonemic	-0.236	-0.59, 0.12	-0.183	-0.54, 0.17
Logical Memory				
Immediate recall	0.164	-0.29, 0.62	0.220	-0.24, 0.68
Delayed recall	-0.137	-0.37, 0.10	-0.106	-0.34, 0.13
**Elevated triglycerides**				
Trail Making Test				
Part A	1.187	-0.05, 2.43	1.183	-0.06, 2.43
Part B	0.720	-1.27, 2.71	0.729	-1.27, 2.73
Verbal Fluency				
Semantic	-0.213	-0.86, 0.43	-0.103	-0.75, 0.54
Phonemic	0.053	-0.35, 0.45	0.091	-0.31, 0.49
Logical Memory				
Immediate recall	-0.473	-0.97, 0.03	-0.425	-0.93, 0.08
Delayed recall	-0.138	-0.40, 0.12	-0.110	-0.37, 0.15
**Low HDL cholesterol**				
Trail Making Test				
Part A	0.909	-0.19, 2.01	0.876	-0.23, 1.99
Part B	0.960	-0.79, 2.71	0.891	-0.88, 2.66
Verbal Fluency				
Semantic	-0.618*	-1.18, -0.06	-0.491	-1.06, 0.08
Phonemic	-0.167	-0.52, 0.18	-0.097	-0.45, 0.25
Logical Memory				
Immediate recall	-0.436	-0.88, 0.01	-0.366	-0.81, 0.08
Delayed recall	-0.264*	-0.49, -0.03	-0.229	-0.46, 0.00
**High blood pressure**				
Trail Making Test				
Part A	0.485	-0.72, 1.69	0.487	-0.72, 1.70
Part B	-0.387	-2.31, 1.54	-0.435	-2.36, 1.49

Verbal Fluency				
Semantic	-0.201	-0.82, 0.42	-0.119	-0.74, 0.50
Phonemic	-0.105	-0.49, 0.28	-0.056	-0.44, 0.33
Logical Memory				
Immediate recall	-0.162	-0.65, 0.32	-0.119	-0.61, 0.37
Delayed recall	-0.177	-0.43, 0.08	-0.150	-0.40, 0.10
**Elevated fasting glucose**				
Trail Making Test				
Part A	1.657	0.10, 3.42	1.600	-0.17, 3.37
Part B	0.814	-2.00, 3.63	0.809	-2.03, 3.65
Verbal Fluency				
Semantic	0.599	-0.31, 1.51	0.761	-0.15, 1.68
Phonemic	-0.062	-0.63, 0.50	0.009	-0.56, 0.58
Logical Memory				
Immediate recall	-0.903*	-1.61, -0.20	-0.865*	-1.58, -0.16
Delayed recall	-0.272	-0.64, 0.10	-0.234	-0.60, 0.14

Abbreviation: CI, confidence interval.

* significant at $p<0.05$

[a] Adjusted for age (continuous), sex, education (primary and secondary school, high school, higher education). [b] Adjusted for age (continuous), sex, education (primary and secondary school, high school, higher education), cardiovascular disease (absence or presence of stroke or ischemic heart disease or heart failure or other heart disease diagnosis), alcohol consumption (never, less than once/month, 1-3 times/month, 1-2 times/week, almost every day) and physical activity (continuous).
